# Status Epilepticus Manifested as Continuous Epileptic Spasms

**DOI:** 10.3389/fneur.2020.00065

**Published:** 2020-02-14

**Authors:** Jianxiang Liao, Tieshuan Huang, Myriam Srour, Yuhan Xiao, Yan Chen, Sufang Lin, Li Chen, Yan Hu, Lina Men, Jialun Wen, Bing Li, Feiqiu Wen, Lan Xiong

**Affiliations:** ^1^Shenzhen Children's Hospital Affiliated With China Medical University, Shenzhen, China; ^2^Montreal Children's Hospital, McGill University, Montreal, QC, Canada; ^3^Montreal Neurological Institute and Hospital, McGill University, Montreal, QC, Canada

**Keywords:** status epilepticus, epilepsy, continuous epileptic spasms, electroencephalograms, electro-clinical epilepsy syndrome, video-EEG

## Abstract

**Objective:** The etiology and outcome of status epilepticus with continuous epileptic spasms have not been fully understood; and only rare cases have been reported in the literature. Here, we described 11 children, who manifested continuous epileptic spasms with various etiologies and different outcomes.

**Methods:** This is a case series study designed to systematically review the charts, video-electroencephalography (video-EEG), magnetic resonance images, and longitudinal follow-up of patients who presented continuous epileptic spasms lasting more than 30 min.

**Results:** Median age at onset was 2 years old, ranging from 2 months to 5.6 years. The etiology of continuous epileptic spasms for these 11 cases consisted of not only some known electro-clinical epilepsy syndromes like West Syndrome and Ohtahara Syndrome, but also secondary symptomatic continuous epileptic spasms, caused by acute encephalitis or encephalopathy, which extends the etiological spectrum of continuous epileptic spasms. The most characteristic feature of these 11 cases was prolonged epileptic spasms, lasting for a median of 13.00 days (95% CI: 7.26–128.22 days). The interictal EEG findings typically manifested as hypsarrhythmia or its variants, including burst suppression. Hospital stays were much longer in acute symptomatic cases than in primary epileptic syndromic cases (59.67 ± 50.82 vs. 15.00 ± 1.41 days). However, the long-term outcomes were extremely poor in the patients with defined electro-clinical epilepsy syndromes, including severe motor and intellectual developmental deficits (follow-up of 4.94 ± 1.56 years), despite early diagnosis and treatment. Continuous epileptic spasms were refractory to corticosteroids, immuno-modulation or immunosuppressive therapies, and ketogenic diet.

**Conclusion:** Continuous epileptic spasms were associated with severe brain impairments in patients with electro-clinical syndromes; and required long hospital stays in patients with acute symptomatic causes. We suggest to include continuous epileptic spasms in the international classification of status epilepticus, as a special form. Further investigations are required to better recognize this condition, better understand the etiology, as well as to explore more effective treatments to improve outcomes.

## Introduction

Epileptic spasms were recognized as a type of seizure by the International League against Epilepsy (ILAE) seizure classification in 2001 (1). Spasms typically last 1–2 s and can occur in clusters or in isolation (2). The ictal electroencephalogram (EEG) usually shows high amplitude slow waves with brief spindle-like fast activity. One of the characteristic features of these clinical epileptic spasms is a brief rhomboid-shape appearance on the surface electromyography (EMG) of the deltoids. Epileptic spasms occur primarily in infants but may also occur in other age groups (3–5), even in adults (6). Epileptic spasms can be the main seizure type of some primary epilepsy syndromes (7, 8), such as infantile spasms, Ohtahara Syndrome, and West Syndrome. Epileptic spasms have also been described in Lennox-Gastaut Syndrome (9), and could be seen in some acute secondary conditions (10, 11). Although burst suppression on EEG is one of the features of Ohtahara Syndrome (12–14), and hypsarrhythmia and its variants are recognized as the characteristic interictal EEG patterns of West Syndrome (15–17), patients with epileptic spasms may not have classic hypsarrhythmia EEG patterns but rather show some periodic and focal patterns (9, 18–20), such as hemispheric hypsarrhythmia (21), or even a lack of hypsarrhythmia on EEG (19). The pathogenesis and pathophysiology of epileptic spasms are mostly unknown, and their treatment remains quite challenging (22, 23). The prognosis of epileptic spasms is generally devastating and is associated with poor neurodevelopmental and behavioral outcomes, particularly in some patients with an underlying genetic etiology such as in CDKL5 encephalopathy, a rare X-linked genetic disorder, and CDKL5 genetic changes or mutations have been found in children diagnosed with infantile spasms, Lennox-Gastaut Syndrome, Rett Syndrome, West Syndrome (12).

In 1986, Dr. David L. Coulter described one case of a 3 1/2 months old boy with continuous epileptic spasms in clusters upon awakening, which were only terminated upon the administration of lorazepam and paraldehyde (24). Dr. Coulter failed to identify the etiology of the continuous epileptic spasms for this patient, despite several targeted examinations and laboratory tests, but he first suggested that continuous spasm attacks lasting over 30 min should be considered as a form of status epilepticus. Only a few similar cases have been reported afterwards (14).

The most recent ILAE classification of status epilepticus in 2015 involves four axes: 1. seizure semiology; 2. etiology; 3. EEG correlates and 4. Age (25). In the current status epilepticus ILAE classification, seizure semiology is divided between non-convulsive status epilepticus and those with prominent motor manifestation, which include convulsive, myoclonic, focal motor, tonic, and hyperkinetic seizures (25). Seizure semiology has been shown to be critical to the prognosis of status epilepticus in general (25). The nature of the etiology could be symptomatic (e.g., stroke, encephalitis), remote (e.g., posttraumatic, postencephalitic), progressive (e.g., brain tumor, Lafora's disease), unknown or cryptogenic (25). However, continuous epileptic spasms are not specifically included in this international classification, most likely due to its rare occurrence and under-recognition. Here, we describe 11 cases who manifested continuous epileptic spasms with various etiologies and report their long-term follow-up outcomes. The purpose of this study is to increase awareness of such cases and their associated causes, as well as the need to identify effective intervention and treatments.

## Methods

In this case series study, we systematically reviewed the available clinical data, including medical charts, video-EEG, magnetic resonance imaging (MRI) and other auxiliary tests in 11 patients, who were admitted to the Shenzhen Children's Hospital from 2014 to 2016, with continuous epileptic spasms lasting 30 min or longer. The study was approved by the Shenzhen Children's Hospital Committee of Medical Ethics. Each patient had at least one video-EEG recording, and each video-EEG included both a waking state and a sleep state of at least one sleep cycle. When epileptic spasms were identified, the duration of the spasms, suppression on EEG, and intervals between spasms were manually measured and calculated. The spasms within an interval of <1 min were defined as being in one cluster (2). The parents and nurses were taught to recognize long-lasting continuous spasms, so when the seizure manifestation appeared or changed, video-EEG was arranged to confirm the continuous spasms. The EEG recordings (Nicolet monitor system, international 10–20 system) were reviewed by at least two experienced pediatric neurologists who were trained and certified in pediatric EEG, and confirmed by a third expert in this field. The etiological causes of the patients' continuous epileptic spasms were defined according to the ILAE task force on classification proposal (26). Statistics regarding the variables of age, duration of epileptic spasms and follow-up periods were descriptive, and variables are presented as the mean ± standard deviation (SD). To compare the clinical variables when the diagnosis of status epileptics of spasms was established, a *t*-test was performed for each comparison and a *P*-value of < 0.05 was accepted as statistically significant.

## Results

Between July 2014 and August 2016, video-EEGs were performed in 3,378 in-patients at the Shenzhen Children's Hospital. The duration of the recordings varied from a minimum of 2.5 h to 15.0 h, including overnight recordings (in 725 patients, 19.60% of recordings). We identified 11 cases with status epilepticus of continuous epileptic spasms, which lasted longer than 30 min. This result represented approximately 3 cases per 1,000 admitted patients for suspected seizures during this period.

The average age at onset of continuous epileptic spasms was 2.57 ± 2.22 years (ranging from 0.14 to 5.58 years, median 2.00 years, 95% CI: 1.08–4.06 years). The duration of the continuous epileptic spasms was 67.74 ± 90.02 days, ranging from 0.02 to 240.00 days, median time 13.00 days (95% CI: 7.26–128.22 days). All the patients also presented with altered consciousness and other types of seizures. The duration of follow-up was 4.94 ± 1.56 years (range: 3.33–9.00 years), with a median follow-up of 4.25 years (95% CI: 3.89–5.59 years). The age of onset was significantly older (at 4.28 ± 1.44 years) in the acute symptomatic cases (Cases 1 to 6, see in Table 1) compared to 0.53 ± 0.4 years in the non-acute non-symptomatic cases (Cases 7–11) (*P* = 0.0001).

**Table 1:** Patients' clinical data and follow-up.

**No. of case** **Age at investigation** **Sex** **Hospital stay**	**Diagnosis**	**Etiology**	**Spasms' duration**	**Other seizure types**	**Brain MRI findings**	**Treatment**	**Follow-up**
Case 1 4 years Male 30 days	Mycoplasma-pneumoniae-related encephalopathy	Mycoplasma pneumoniae infection	Lasting 13 days, burst suppression both in sleep and awake	Focal seizures, originated from right frontal or parietal areas	At Day 15: atrophy of both cerebrum and cerebellum	IVIG, MP, 8 AEDs, KD; at investigation: TPM, LTG, CZP, LEV	At 9 years, walking, but not speaking, could not recognize parents, short stature
							
Case 2 5 years and 2 months Female 37 days	Influenza-virus-type-one-related acute encephalopathy	H1N1 infection	Lasting 6 months, burst suppression, gradually alleviated	Focal seizures, originated bilaterally	Generalized atrophy after decompressive craniectomy	IVIG, MP, KD, prednisone, 4 AEDs	At 5 years and 1 month, walking, mental retardation, no language
							
Case 3 5 years and 7 months Male 16 days	Anti-NMDAR encephalitis	Autoimmune encephalitis	Lasting 8 months, burst suppression EEG	Focal seizures originated right hemisphere, convulsive SE	At 10 months: MRI showed atrophy of cerebrum, and hippocampi improved	IVIG, MP, 5 AEDs; at investigation: VPA, NZP, LTG, CBZ, KD, cyclophosphamide monthly 8 times	At 4 years, nearly recovered, autoantibody remains positive in CSF
							
Case 4 5 years and 7 months Male 105 days	Febrile-infection-related epilepsy syndrome	Unknown etiology, Mycoplasma pneumoniae infection?	Lasting 87.5 min, asymmetrical spasm	Convulsive and non-convulsive SE, multi-focal, primarily frontal in origin	Brain atrophy including hippocampi improved during the last 3 months	IVIG, PE, KD, propofol, ketamine, chloralhydrate, diazepam, 7 AEDs, with 5 AEDs when discharged.	At 4 years, walking, speaking, but memory was impaired
							
Case 5 2 years Male 29 days	Allergenic demyelinating encephalitis	Vaccination of measles-mumps-rubella triad vaccine	Lasting 2 months, each cluster lasting more than 30 min, burst suppression and hypsarrhythmia	Focal seizures, left frontal and temporal, bilateral occipital epileptiform discharges	Atrophy of thalamus, occipital region and left hippocampus improved?	IVIG, MP, 3 AEDs, ACTH	At 4 years, nearly recovered as before, learning and language slightly impaired
							
Case 6 3 years and 4 months Female 141 days	Acute encephalopathy	Severe allergy, post-cardiopulmonary resuscitation, iodine allergy	Lasting 30–60 min daily for 50 days. EEG hypsarrhythmia	Focal seizures generalized at early stage of disease	Caudate nucleus and thalamus abnormal signal on Diffusion-Weighted Images	TPM, ACTH, prednisone	At 4 years 2 months, left arm movement and speech not fully recovered, otherwise normal
Case 7 11 months Female 15 days	Intractable epilepsy, focal seizures, developmental delay	Compound heterozygous mutation in the *TBCD* gene	3 times continuous spasms, each lasing 1.5 weeks	Low voltage on EEG, non-Convulsive status epilepticus, convulsive status epilepticus	At 20 months: significant atrophy, 3 months later atrophy aggravated	IVIG, MP, KD, 3 AEDs, another 3 AEDs used	At 5.5 years, no language, rolling and walking, severe development delay
							
Case 8 5 months Male 13 days	Intractable epilepsy, infantile spasms, Ohtahara Syndrome transition Severe asphyxia	Ohtahara Syndrome, genetic mutation?	Lasing 2 h, burst suppression and hypsarrhythmia	Focal seizures, tonic seizures frequently, Multifocal spikes	Severe brain atrophy	KD, prednisone, 4 AEDs, ACTH	At 4 years 3 months: microcephaly, no head control or language, cannot recognize parents, seizures decreased
							
Case 9 11 months Female 17 days	Infantile spasms, psychomotor retardation, epileptic encephalopathy	Unknown	Lasing 3 months and 5 days, EEG, burst suppression	Focal seizures, refractory epilepsy	Abnormal signal at white matter, thalamus atrophy	TPM, KD, VPA, LEV, ACTH	At 6 years: developmentally delayed, could not sit, still daily seizures
							
Case 10 1 month and 20 days Male 15 days	Ohtahara Syndrome Asphyxia	Genetic, a *de novo* missense mutation in the *KCNQ2* gene	Lasting 108 min, spasms in cluster or singly	Tonic seizures, focal seizures	Un remarkable	ACTH, 3 AEDs	At 3 years and 4 months: spasms, no visual fixation, deceased at age of 8 months due to severe infection.
							
Case 11 4 months Male 15 days	Pachygyria malformation, Intractable epilepsy	Genetic, 489 Kb deletion at 17p13.3	Lasting 30 min, in cluster or single, hypsarrhythmia	Tonic seizures, focal seizures	Pachygyria	3 AEDs, ACTH, sirolimus, KD	At 5 years: severely delayed development, no head control, language or visual following, seizures 3.5 times daily

*IVIG, intravenous immunoglobulin; PE, plasma exchange; MP, methylprednisolone; ACTH, adrenocortical tropical hormone; AEDs, antiepileptic drugs; TPM, topiramate; LTG, lamotrigine; LEV, levoparacetam; NZP, nitrozepam; CBZ, carbamazepine; KD, ketogenic diet*.

The etiologies identified in the acute symptomatic spasm patients were diverse, included combined enterovirus type 71 and mycoplasma-pneumonia-related encephalopathy (Case 1), influenza-virus-type-A-related encephalopathy (Case 2), anti-N-methyl-D-aspartate receptor (anti-NMDAR) encephalitis (Case 3), febrile-infection-related epilepsy syndrome (FIRES, Case 4), acute post-vaccination encephalopathy from the measles-mumps-rubella vaccine (Case 5), and acute anaphylaxis from iodine disinfectant at the beginning of a tooth surgery (Case 6). The etiologies in the non-acute cases, mostly with established electro-clinical syndrome cases were also heterogenous, including compound heterozygous mutations in the *TBCD* gene (ENST00000355528, c.881G>A, p.Arg294Gln and c.22801C>A, p.Tyr760^*^, Case 7), severe perinatal asphyxia (Case 8), a *de novo* heterozygous missense mutation in *KCNQ2* (ENST00000359125, c.1657C>T, p.Arg553Trp) in a patient with Ohtahara Syndrome (Case 10), one case of pachygyria malformation associated with a pathogenic *de novo* chromosomal copy number variation (489 Kb deletion at 17p13.3, chr17: 2441590-2931074 [hg19], Case 11); with only one case with unknown etiology (Case 9) (see also Table 1).

We compared different categories of cases among our series, and with the typical cases epileptic spasms in our hospital, and identified the following characteristic features in our cases. The hospital stays of the acute cases (Case 1 to Case 6) were significantly longer than the ones with primary epileptic syndromes (Case 7, Case 10 and Case 11) (59.67 ± 50.82 vs. 15.00 ± 1.41 days, *P* = 0.0299), while the average hospital-stay in our department during the same time period was < 7 days. The primary epilepsy syndromic cases (Case 7, 10, 11) had earlier onset of seizures, and their conditions progressively deteriorated with unresolved seizures. Neurodevelopmental and seizure outcomes after 4.94 ± 1.56 years, based on clinical interview and parental reporting, were much better in the 6 cases with acute symptomatic epileptic spasms than in the five cases with an established electro-clinical syndrome, which were mostly of genetic origin, despite the absence of any significant difference in the duration of continuous epileptic spasms between the two groups (*P* = 0.1885). Cases 7–11 had more severe global developmental delay, including severe intellectual deficiency, language and movement impairment; and epileptic spasms were still present at the last follow-up in all cases with epileptic syndromes. In addition, one patient (Case 8) died of severe infection at age of 8 months. Our results indicate that etiology might be an important factor in determining the outcome of the patient's epilepsy and neurodevelopment.

**Figure 1:**
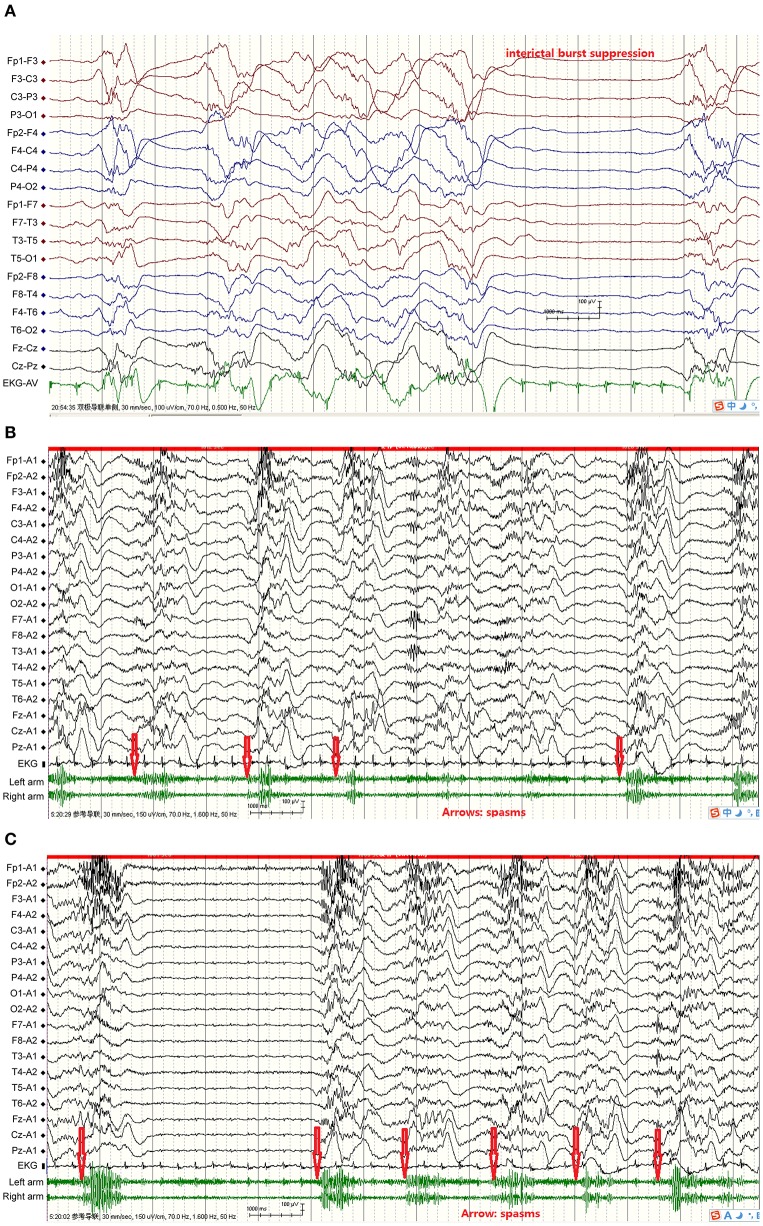
From Case 4 showed FIRES, acute symptomatic continuous epileptic spasms, interictal hypsarrhythmia and suppression-burst pattern, and ictal EEG, plus EKG, and EMG. **(A)** Interictal burst suppression, bursts 1–5 s, inhibition 1–4 s; **(B)** Spasms simultaneous EEG showed fast wave, then slow wave followed by voltage attenuation, spasms synchronized with initial fast waves. Red arrows indicate spasms initiation; **(C)** Spasms simultaneous EEG showed fast wave, then slow wave followed by voltage attenuation, spasms synchronized with initial fast waves; two slow waves consecutively appeared in spasms 1, 2, 3, and 5. Spasms more frequent and heart rate faster than interictal stage. Red arrows indicate spasms initiation.

The duration of the continuous epileptic spasms ranged from 30 min to 8 months according the video-EEG recordings and the patients' seizures diaries. The attacks manifested distinctly upon arousal from sleep and decreased or disappeared during sleep. Some of the spasms were asymmetrical or weak with mild frowning and tremor of body or limbs. The inter-spasm intervals were generally longer than 1 s. The intervals between the spasm attacks varied between the patients, but also varied from one attack to another for the same individual. Ictal EEGs during the 1–2 s of spams showed mostly fast waves followed by a single high amplitude slow wave, and then voltage attenuation. In the case with Ohtahara Syndrome, the ictal EEG showed spasm attacks nearly synchronized with the burst of suppression phase (Figure 1). In the rest patients, the interictal EEGs showed hypsarrhythmia or its variants, i.e., burst suppression or periodic hypsarrhythmia pattern; and the EEG background was often generalized or multifocal slow waves.

The continuous epileptic spasms in our patients were generally resistant to multiple anticonvulsants (Table 1); and the outcomes were worse in the patients with continuous spasms mixed with other types of seizures, including focal seizures, tonic seizures etc. Immunotherapy was used in all patients, and treatments included intravenous immunoglobulin (6 cases), high doses of methylprednisolone (5–15 mg/kg/day) (3 cases), and immunosuppressors (such as cyclophosphamide in one case), but with no satisfactory results.

## Discussion

We report a series of 11 cases with continuous epileptic spasms as a severe form of status epilepticus. Their clinical characteristics included: (1) each epileptic spasm episode lasted longer than 30 min though the seizure semiology was similar to the classical epileptic spasms; (2) etiologies were diverse, including acute symptomatic causes such as encephalitis, encephalopathy, and FIRES; even in the cases with known electro-clinical epilepsy syndromes showing different genetic mutations; (3) the disease course and hospital-stay in acute symptomatic cases were much longer than in those with known electro-clinical epilepsy syndromes; (4) the cases with the established electro-clinical syndromes showed the worst outcome, despite earlier diagnosis and interventions.

Although continuous epileptic spasms have only been rarely described in the literature, our experience indicate that careful review of video-EEG and prudent observation from the parents and health professionals were critical for detecting continuous epileptic spasms. Our series showed a recognizable epileptic spasms semiology pattern, which fits with the ILAE definition of status epilepticus (25). The underlying etiologies seem to be diverse, including infection, and/or inflammatory-mediated processes and genetic disorders, or still unknown causes. The etiological heterogeneity of continuous epileptic spasms is similar to those with convulsive status epilepticus, occurring in the similar age groups, including both acute encephalitis or encephalopathy and other symptomatic spasms associated with defined electro-clinical syndromes, such as the Ohtahara Syndrome and West Syndrome, which are mostly with genetic origin; and the patients often exhibited additional types of seizures, such as focal seizures, with or without generalization. Our results suggest that the long lasting spasms accompanied with disturbance of consciousness and other types of seizures could predict long hospital-stay and much worse neurodevelopmental outcomes than those with epileptic spasms lasting <30 min. The outcome was consistently poor in patients with electro-clinical epilepsy syndrome despite early recognition and treatment, which seems to be different from classical West Syndrome or other epileptic encephalopathies in which early recognition and treatment leads to improved neurologic outcomes.

The recognition and diagnosis of continuous epileptic spasms is challenging, particularly in acutely and critically ill children when first admitted to the pediatric intensive care unit (PICU). The epileptic spasms are not as easily noticeable as focal motor seizures and generalized tonic-clonic seizures. Lack of clinical experience, inadequate observation and short video-EEG monitoring may result in misdiagnosis or underdiagnosis. In our study, the patients were sometimes in a supine position and were so weak that their movements manifesting epileptic spasms were quite subtle, therefore easily overlooked by the observers. At times, the subtle spasm movements could also be misinterpreted as extra-pyramidal motor dyskinesia. In addition, the patients often had co-occurring focal motor seizures or tonic-clonic seizures so the faint epileptic spasms were unnoticed.

Hypsarrhythmia is a characteristic interictal EEG feature of epileptic spasms (15–17). This condition emerges gradually and can be used to predict the onset of infantile spasms or the recurrence of spasms after discontinuing adrenocorticotropic hormone (ACTH) administration (27, 28). However, the classic EEG pattern of hypsarrythmia is recorded in fewer than half of cases of epileptic spasms; instead, variants similar to paroxysmal or periodic hypsarrhythmia have been recorded and such variants of hypsarrhythmia may be as high as 69% (16), which could easily be overlooked or misinterpreted.

Continuous video EEG monitoring has been recommended for at least 24 h in critically ill or complex cases, who are resistant to initial treatment, to rule out the presence of status epilepticus of epileptic spasms or non-convulsive status epilepticus (26, 29, 30). Physicians, nurses, or EEG technicians trained in cerebral function monitoring must be on-site to observe and detect possible epileptic spasms or other seizures types since long-lasting seizures or epileptiform discharges may cause brain damage and result in poor prognoses (26). Early diagnosis is extremely important so that early and aggressive intervention could be undertaken to improve outcomes and prognosis, especially in children with acute symptomatic spasms status epilepticus.

Among all the currently available therapies, ACTH and vigabatrin have the best response rate in infantile spasms (31–35). Anecdotal reports and animal experiments indicate that lorazepam, clobazam, paraldehyde, ACTH, and other corticosteroids may be effective in the treatment of continuous epileptic spasms (27). Our preliminary experience indicated that acute symptomatic spasms could also be treated similarly to autoimmune encephalitis (36, 37), which includes ACTH, intravenous immunoglobulin, high doses of methylprednisolone, immunosuppressors such as cyclophosphamide and rapamycin (38), and a ketogenic diet. Acute symptomatic cases seemed to have responded better to immuno-therapy.

Obviously, our study was limited by the small sample size and the nature of a retrospective study. Future prospective studies with larger sample size, and/or randomized, controlled, multi-center clinical trials could help to further characterize this particular type of status epilepticus, for example, to quantify precisely the duration of epileptic spasms, the number of spasms in each cluster attack, the statistical cutoff for continuous epileptic spasms as status epilepticus, as well as age and video-EEG and their correlation with the prognosis.

In summary, continuous epileptic spasms is most likely a clinically distinct form of status epilepticus. Etiologies include severe acute encephalopathy, encephalitis or FIRES in acute symptomatic conditions, as well as various genetic causes in defined electro-clinical syndromes. The acute symptomatic cases often need longer hospital-stay, and usually have changes in level of consciousness and other types of seizures. However, the cases with known electro-clinical epileptic syndromes showed the worst long-term outcome. All the cases were generally resistant to conventional AEDs and ACTH treatments; therefore, more effective treatment is urgently needed. We suggest that continuous epileptic spasms be considered to be included into the classification of ILAE status epilepticus in the future.

## Data Availability Statement

The datasets generated for this study are available on request to the corresponding author.

## Ethics Statement

The studies involving human participants were reviewed and approved by Ethics Committee of Shenzhen Children's Hospital. Written informed consent from the participants' legal guardian/next of kin was not required to participate in this study in accordance with the national legislation and the institutional requirements.

## Author Contributions

JL, TH, YC, and YX were responsible for EEG reviewing. JL, MS, and LX were responsible for the design and completion of the manuscript and figures. All authors were responsible for the acquisition and analysis of data.

### Conflict of Interest

The authors declare that the research was conducted in the absence of any commercial or financial relationships that could be construed as a potential conflict of interest.
